# Beyond the denial of death: death meditation increases a sense of connectedness and appreciation of life

**DOI:** 10.3389/fpsyt.2024.1477479

**Published:** 2024-11-28

**Authors:** Enny Das, Marieke L. Fransen, Mary Beth Oliver

**Affiliations:** ^1^ Centre for Language Studies, Radboud University, Nijmegen, Netherlands; ^2^ Behavioral Science Institute, Radboud University, Nijmegen, Netherlands; ^3^ Belissario College of Communications, The Pennsylvania State University, University Park, PA, United States

**Keywords:** death, art, mortality salience, eudaimonia, connectedness, life appreciation

## Abstract

**Introduction:**

The pervasive denial of death in modern society has created an unbalanced relationship with death that gets in the way of living a full life. To address this problem, the Lancet Commission on the Value of Death recently proposed principles of a healthier scenario for the future. In this ‘realistic utopia’, death is recognized as having value, and conversations about death and dying have become common. The present research examined if art could help to decrease death denial and increase life appreciation.

**Methods:**

An art installation invited visitors to confront thoughts of their own death in a guided meditation by visualizing the decay of their own dead body. Visitors’ responses were compared to those of a standard death reflection group and a neutral control group (*N*=229) at two time points. Feelings of being moved, connectedness to a higher power, and life appreciation were assessed immediately (T1); death reflection and life appreciation were assessed two weeks later (T2) (N=105).

**Results:**

At T1, the art installation induced higher levels of being moved and connectedness to a higher power than the two control groups. At T2, the art installation induced more lingering reflection than the two control groups. Lingering reflection, in turn, increased appreciation of life.

**Discussion:**

We show that art can be harnessed to promote a more balanced relationship with death, and greater appreciation of life. The art installation provided individuals with concrete, and more encompassing simulations of what death could be like. By placing death in this bigger perspective, the art installation encouraged conscious death reflection. Such a connected perspective is often lacking, but direly needed, in healthcare and in larger society.

## Introduction

“Remembering death keeps us awake, focused, and ready for whatever might happen — both the excruciatingly difficult and the breathtakingly beautiful.”

- Theresa Aletheia Noble (1)

In modern society, death and dying have become unfamiliar, and skills, traditions, and knowledge related to death are being lost (2). The denial of death (3) has created an unbalanced relationship with death - treating it as an existential problem that should be avoided rather than a natural part of the life cycle that should be discussed. To change this situation, the Lancet Commission on the Value of Death recently proposed five principles of a realistic utopia (2), in which death is recognized as having value and conversations and stories about everyday death, dying, and grief becoming more common.

Standing in the way of meaningful conversations about death and dying is human’s unconscious tendency to suppress thoughts about death and dying. Decades of research on Terror Management Theory (4) have shown that human’s typical response to reminders of their mortality is to adhere to cultural belief systems that move the thought of death outside of conscious awareness (5). This points to the need to find effective strategies that stimulate meaningful death conversations without evoking resistance, suppression, or denial.

Ancient Buddhist and philosophical traditions propose that practicing the art of dying helps to decrease the denial of death and increase one’s appreciation for life. In the so-called Nine Cemetery Contemplations, individuals focus on visualizing the nine stages of decay of their own body (6). In ancient times, Buddhists would engage in this practice for days while seated next to a corpse, but this practice became obsolete when hygienic laws became stricter. In modern society religious practices are on the decline, but the need to make sense of death remains.

Recently two artists created a secular, adapted version of the original Nine Cemetery Contemplations in an art installation entitled ‘This body that once was you’ (7). Visitors of the art installation were asked to visualize the nine stages of decay of their own bodies in a guided audio meditation. Seated in the middle of a field of aerosolized human bone dust in a large concert hall, they listened to a carefully crafted narrative about their own demise, starting with the sight of one’s own dead body (first stage), and ending in the sight of one’s own bone dust being scattered by the wind, becoming recycled into other life forms (final stage). We worked with these artists to empirically test sustained effects of their art installation on visitors in a field experiment. This article reports on the findings and reflects on the implications for healthcare practice and larger society, building on psychological, communication, and medical research relevant to the topic of death.

### Death meditation as a meaningful experience

A wealth of research shows that humans typically push death thoughts outside of conscious awareness to avoid becoming paralyzed by the fundamental fear of death. To achieve this, they use an anxiety buffer that consists of two components: a belief in the validity of one’s cultural worldview and its associated standards, and a belief that one is meeting cultural standards, which serves to heighten self-esteem (8). In the past decades, hundreds of studies have provided support for these premises, showing that adhering to cultural belief systems suppresses active death thoughts (5).

Even though humans typically prefer to avoid or suppress death thoughts, they are in fact capable of approaching and transcending this fear, provided that the right circumstances are met. The Terror Management Theory of Meaningful Entertainment (TMT-ME) (9) proposes that especially meaningful entertainment experiences about death, such as watching a movie in which a main character dies, can invite viewers to confront the fear of death at a safe aesthetic distance. These effects occur especially when viewers’ fundamental fear of death is salient in real life, and the entertainment portrays the death as meaningful. Building on these findings, we propose that the simulated experience offered by contemplations about death in an art installation can be conceptualized as a eudaimonic entertainment experience, which allows visitors to try out and confront thoughts about death at a safe aesthetic distance, provided that their fear of death is salient, and that the simulated death is perceived as meaningful.

Two key processes play an important role in whether people assign meaning to sad or tragic experiences: reflective thoughts and feeling moved or inspired emotions (10). First, meaningful experiences evoke mixed emotions like the emotion of being moved (11), or the co-occurrence of positive and negative affect (12). The art installation tested in the present research asks visitors to face the terrifying fact that they, too, are mortal beings, which is likely to trigger negative emotions. However, it also provides visitors with positive experiences, such as the sense of beauty of being all alone in a large concert hall, the poetic words used in the guided meditation, and the meaningful ‘material incarnation’ ending in which visitors’ imagine bone dust becomes part of new life forms. We therefore expected that individuals who were exposed to the art-induced death reflection experience would show higher levels of being moved, compared with individuals who were asked to reflect on their own death without the help of the art installation, or individuals who did not reflect on death at all (H1).

Apart from the emotion of being moved, meaningful entertainment experiences are typically associated with higher levels of reflective processing (13). Meaningful death portrayals may make people reflect on death and think about what it means to them. In this way, reflective thought can play a crucial role in eliciting self-transcendence, not only during the experience, but also afterwards, in so-called lingering reflections that occur after completion of an experience. This process can be compared to the process of retrospective imaginative involvement (RII) (14), i.e., thinking about a story character after completion of a narrative experience. However, unlike RII, lingering reflection pertains to thinking back on one’s own experience, rather than the vicarious experience of identifying with a story character, after the experience ended. We therefore expected that individuals who were exposed to the art-induced death reflection experience would show higher levels of lingering reflection, compared with individuals who were asked to reflect on their own death without the help of the art installation, or individuals who did not reflect on death at all (H2).

### Death transcendence

Can simulated experiences with death evoke death transcendence, including a fresh outlook on life? Research on near death experiences (NDE) suggests that this may indeed be the case. Individuals who have experienced a real or simulated NDE typically report increased connectedness with fellow humans, with nature and a higher power (15), and posttraumatic growth, such as an increased appreciation of life (16). What is striking about these findings is that NDE appear to offer a worldview transcendence rather than cultural worldview defense strategies that are typically observed after mortality salience (MS) manipulations in the lab. Research on simulated NDE’s in an experimental setting had effects comparable to those of actual NDEs, yet diametrically opposed to a typical MS manipulation (17). Building on these findings, we expect that individuals who were exposed to the art-induced death reflection experience would show higher levels of connectedness (H3a) and life appreciation (H3b), compared with individuals who were asked to reflect on their own death without the help of the art installation, or individuals who did not reflect on death at all. We expected these death transcendence effects to sustain over time.

## Method

### Design and participants

G-power was used to calculate the sample size for our study. The analysis was conducted for an ANOVA with three groups with a power of.80 and an effect size of *f* =.25. The effect size was based on previous meta-analyses on mortality salience effects demonstrating a small-to-medium effect of *g* = 0.34 (18) and moderate effects (*r* = .35) (5). The analysis revealed that 158 participants were needed to obtain the desired power. In total 229 individuals participated in the research, evenly distributed across the three groups in a 3 (visualization exercise: art installation vs. death control vs. neutral control) between-subjects design. The experimental group consisted of all visitors of the art installation. Remaining participants were randomly assigned to the two control groups.

Participants’ mean age was 49.15 (*SD*=15.86). The majority was female (*N* = 166, 72.5%). Most participants had completed a higher education; 53% had a master’s degree; 36.7% a bachelor’s degree; the remainder had completed high school or lower vocational training (8.3%) or another form of education (1.3%). The majority (92.6%) reported personal experience with loss. Age was distributed equally across experimental groups, *F*(2, 126)=2.07, *p*=.129), as was gender (*χ* ^2^(4)=4.59, p=.332), education (*χ* ^2^(8)=6.02, *p*=.656), and personal experience with loss (*χ* ^2^(2)=1.72, p=.422). We also assessed participants’ self-esteem, which plays an important role in TMT research, and whether they were spiritual. Participants scored average on self-reported spirituality (*M*=3.72, *SD*=1.95 on a 7-point scale), with no differences between conditions *F*(2, 126)=1.18, *p*=.310). Self-esteem was moderate (*M*=4.75, *SD*=1.25 on a 7-point scale), with no differences between conditions *F*(2, 126)=1.02, *p*=.361; See **Table 1**.

At T2 (N=105), the equal distribution of demographic variables across conditions was retained for all relevant demographic characteristics, i.e., age, gender, education, spirituality, self-esteem and loss of a loved one (See **Table 1**).

**Table 1:** Distribution of participant characteristics across experimental conditions at T1 and T2.

T1 (N=229)	Art installation	Death control	Neutral control	p’s
Age	46 (15)	51 (17)	50 (15)	.129
Female %	71.3	71.2	75.0	.332
Highest completed education: Highschool Lower vocational Higher vocational University Other	2 (2.5%) 2 (2.5%) 27 (32.1%) 49 (39.8%) 0 (0.0%)	3 (4.1%) 5 (6.8%) 29 (34.5%) 34 (27.6%) 2 (2.7%)	3 (3.9%) 4 (5.3%) 28 (33.3%) 40 (32.5%) 1 (1.3%)	.656
Spirituality	3.94 (1.98)	3.74 (1.99)	3.46 (1.86)	.310
Self-esteem	4.63 (1.28)	4.73 (1.20)	4.91 (1.26)	.361
Lost a loved one	73 (91.3%)	70 (95.9%)	69 (90.8%)	.422
T2 (N=105)	Art installation	Death control	Neutral control	p’s
Age	47 (15)	54 (17)	54 (15)	.069
Female %	71.2	65.4	63.0	.459
Highest completed education: Highschool Lower vocational Higher vocational University Other	0 (0.0%) 1 (1.9%) 20 (38.5%) 31 (59.6%) 0 (0.0%)	2 (7.7%) 2 (7.7%) 12 (46.2%) 9 (34.6%) 1 (3.8%)	0 (0.0%) 2 (7.4%) 10 (37.0%) 15 (55.6%) 0 (0.0%)	.101
Spirituality	3.83 (2.11)	3.77 (2.03)	3.63 (1.84)	.919
Self-esteem	4.52 (1.34)	4.85 (1.05)	4.63 (1.36)	.580
Lost a loved one	49 (94.2%)	24 (92.3%)	27 (100%)	.376

NB. For age, spirituality and self-esteem, means (SDs) are reported, using ANOVA. For gender, education, and experience with loss, percentages are reported within conditions, using crosstabs. Scale range for self-esteem and spirituality is 1-7.

### Procedure and materials

#### Visualization exercise

To keep questionnaire instructions constant across experimental conditions, we used a word that would apply to all three conditions and that we could refer to in the second questionnaire at T2 to summarize the experimental condition participants had been assigned to at T1. This is why we made sure that the label ‘visualization exercise’ was mentioned for all experimental conditions. Specifically, the instruction in the control conditions was formulated as follows: “Visualization exercise: Please write down the emotions you feel when you reflect on your own death/watching TV’ for the death control and neutral control conditions, respectively. In the experimental conditions, the guided meditation voice asked participants to visualize their own dead body.

**Figure 1:**
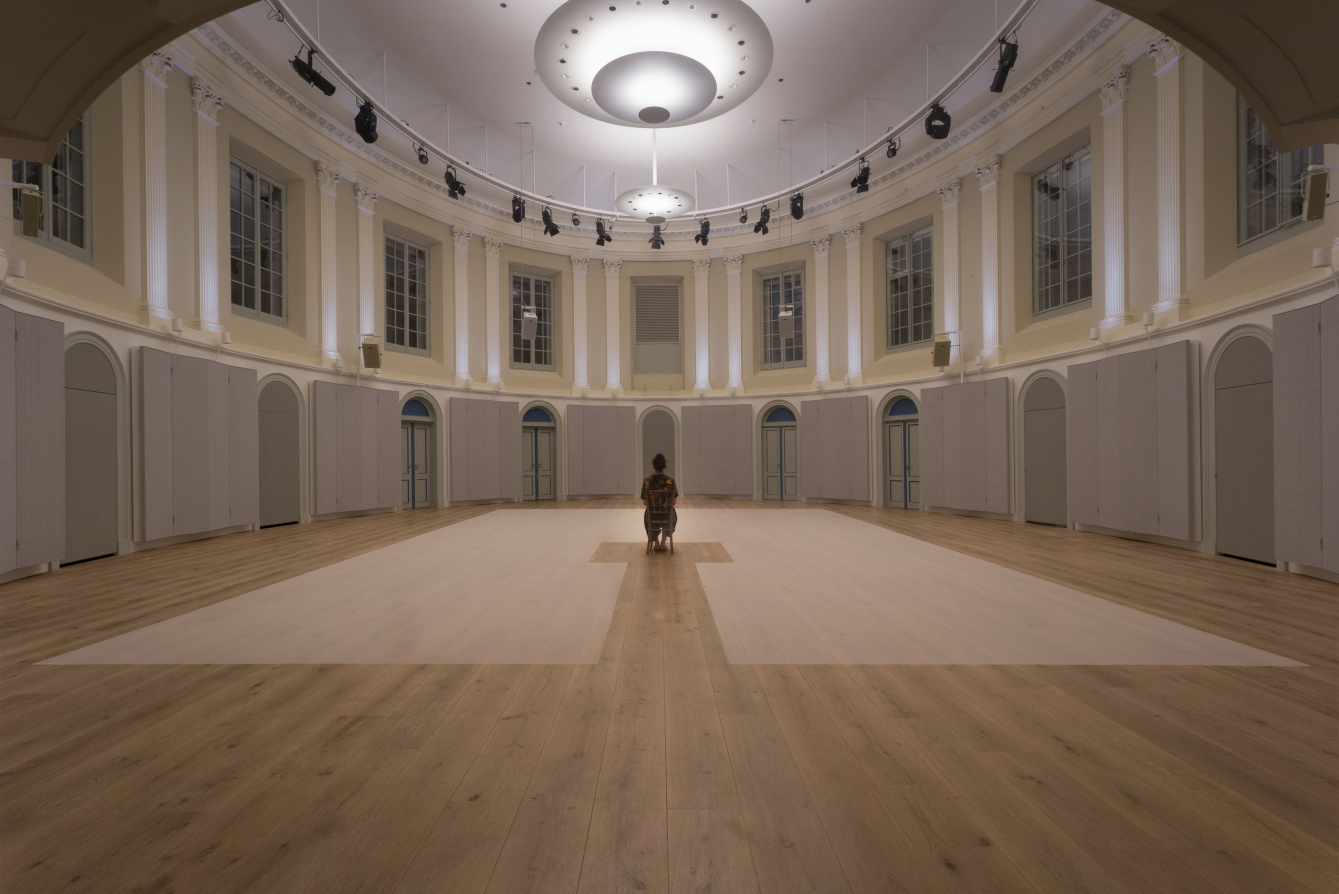
Picture of the art installation. Credits: Ilya Rabinovich.

After informed consent, participants in the art installation condition were guided to a chair in the middle of a field of aerosolized human bone dust in a large concert hall (see **Figure 1**), after which the confederate left, and the participant was alone. When a participant was settled, a technician would start the guided meditation that was broadcast through audio speakers in the concert hall. The guided meditation started with the words: “This is a meditation where you are actually observing your corpse. You are not your corpse. You’re observing it.” In the next 30 minutes, the meditation asked participants to visualize the nine stages of decay of their own corpse in a carefully crafted narrative. The narrative started with the sight of one’s own dead body (first stage) and ended in the sight of one’s own bone dust being scattered by the wind, becoming recycled into other life forms (final stage).

All participants remained seated during this half hour. At the end of the guided meditation, the confederate entered the hall to guide the participant to another room to fill out the questionnaire and debrief them. It was made sure first that the participant felt at ease by asking them how they had experienced the art installation. All participants completed the guided meditation and the subsequently presented questionnaire.

For the death control group, the classic mortality salience manipulation from Terror Management studies (4) was employed, asking participants to: “Please describe the emotions the thought of your own death arouses in you”; “Jot down, as specifically as you can, what you think will happen to you physically as you die and once you are physically dead.” Participants in the neutral control group were asked to describe the emotions and what they think happens to them when they watch television (19). This is a control condition that is used often in TMT research.

#### Procedure

This study was approved by the University’s ethics committee (# 2020-8854). Participants were recruited via regular and social media channels of the art foundation, a national public broadcasting agency, and a national newspaper to take part in the art-meets-science project, an umbrella term we used to cover all conditions of the experimental study. An announcement on the event website mentioned that interested individuals could contribute to the art-meets-science project by first visiting the art installation and then contributing to the scientific study, or by immediately contributing to the scientific study in case they could not make it to the art installation. This ensured that the recruitment process was the same for all conditions. Tickets for the art installation sold out quickly (10 Euros per ticket); people on the wait list were reminded that they could still take part in one of the two control groups. As a compensation, all control participants were offered a limited edition “Memento Mori” birthday calendar as a thank you for their contribution to the art-meets-science project.

Participation was voluntary; individuals who were interested in participating could indicate their interest online and then proceeded to one of the experimental conditions. Upon entering the research, all participants first provided written informed consent. Participants in the experimental group then proceeded to experience the installation on location, and control participants were randomly assigned to one of two control groups.

Participants in all conditions were primed with information about the art installation ‘to experience your own death,’ to make sure that death was equally salient upon entry and to prevent selection effects. Participants filled out demographic and control variables, were then exposed to the visualization exercise that varied per experimental condition, filled out the T1 questionnaire that assessed being moved, negative emotions, absorption, death thought accessibility, connectedness and appreciation of life respectively, were thanked for their participation, and invited to take part in a 1-minute follow-up questionnaire. Participants who had agreed received a second questionnaire that assessed lingering reflection and appreciation of life (second measurement) two weeks later, administered via email.

### Measures

#### Key dependent measures

At T1, 6 items on a 7-point scale measured the extent to which the visualization exercise elicited emotions of being moved, e.g., “I was inspired/moved by the visualization exercise” (1=totally disagree, 7=totally agree; Cronbach’s α= .92) (9). Connectedness was assessed with the subscale ‘connectedness with a higher power’ (12 items, 5-point scale) (20), e.g., ‘The visualization exercise made all things appear to be part of a larger whole’ (1=disagree, 5=agree; Cronbach’s α=.93). Lingering reflection was assessed at T2, two weeks later, to rule out the alternative hypothesis that individuals’ positive responses immediately after exposure to the experimental conditions are merely a form of worldview defense, i.e., a defensive response with the goal to push thoughts of death outside of conscious awareness. We therefore set out to capture more sustained reflective thoughts, building the concept of retrospective imaginative involvement (RII) from Slater and colleagues (14). In essence, RII refers to thinking about a story character after completion of the narrative experience. However, unlike RII measure’s focus on the viewer’s responses to the characters (e.g., imagining the characters in different stories or situations), our measure of reflection specifically focused on participants’ own experience during the art-meets-science project, by asking participants to answer the following question on a 5-point scale, “How often did you think about the art-meets-science project in the past week?” (1=never, 5=many times).

We explicitly and consistently referred to the ‘art-meets-science’ project across all three experimental conditions in order to keep questionnaire instructions constant and therefore comparable across experimental conditions. While the control participants did not visit the installation, it was made clear to them that they were still part of the art-meets-science project.

Appreciation of life was assessed two times, at T1 and at T2 (two weeks later), with the 3-item subscale from the Posttraumatic Growth questionnaire (21). Participants indicated on a 5-point scale to what extent the visualization exercise had e.g., induced a greater appreciation for the value of their own life (Cronbach α=.89;.90; 1=did not experience this as a result of the visualization exercise; 5= experienced this to a great degree as a result of the visualization exercise).

#### Control measures

As a control, two items assessed to what extent the different visualization exercises across conditions evoked different levels of mental simulation on a 7-point scale, e.g. “While visualizing, my attention was absorbed by the images I saw in front of me” (1=totally disagree, 7=totally agree; r=.70) (22). As a control, four items on a 7-point scale assessed negative emotions experienced during the visualization exercise (i.e., anger, disgust, fear, sadness; 1=totally disagree, 7=totally agree; Cronbach’s α= .77). To check for differences in fear of death across conditions, death thought accessibility was assessed with a word completion task consisting of 17 neutral words and 8 words that could be filled out as a death-related word, e.g. coff_ _ [coffee, coffin] (19).

We also included spirituality/religiousness, self-esteem and personal experience with loss as potential confounds. We used 1-item measures to avoid an overly lengthy questionnaire, i.e., “I have high self-esteem” (7-point scale; 1=totally disagree, 7=totally agree) (23), “I see myself as a religious or spiritual person” (7-point scale; 1=totally disagree, 7=totally agree), and “Did you ever lose a loved one?” (yes, no).

#### Data and analyses

Main analyses were conducted with analyses of variance. When a control variable showed differences between conditions, we ran the main analyses with and without this variable to check the robustness of the findings. Indirect effects were tested with the PROCESS macro, using Helmert contrasts to test direct and indirect effects of specific conditions (Model 4) (24). We used the guidelines from Hayes (25) for the repeated measures analyses across T1 and T2, which recommend modeling later measurements while using earlier measurements (aka “lags”) as covariates if you want to predict how a variable score changes over time as a result of an independent variable.

#### Open practice statement

Datafiile and syntax can be found at this DOI: 10.34973/hw0z-rd91.

The doi for these files will be made publicly available for other researchers upon publication of the manuscript. This research was not preregistered.

## Results

### Control analyses

A significant difference in mental simulation was observed between conditions, *F*(2, 226)=15.76, *p*<.001, η*_p_* ^2^=.122. The art installation (*M*=4.50, *SD*=1.46) elicited higher levels of simulation compared to the death control (*M*=3.67, *SD*=1.68; *p*<.001) and the neutral control conditions (*M*=3.10, *SD*=1.55, *p*<.001). The two control conditions also differed (*p*=.027). The art installation did not elicit more negative emotions (i.e., anger, disgust, fear, sadness) than the two control conditions, *F*(2, 226)=1.58, *p*=.209 (*M*=1.97, *SD*=1.07), and there were no differences between conditions on the number of death words, *F*(2,226)=.872, *p*=.419 (*M*=3.70, *SD*=1.81). Thus, the experimental condition did not differentially affect the fear of death and negative emotions but did increase mental simulation. As a robustness check, we conducted the main analyses with and without mental simulation as a covariate.

### T1 measurements

The ANOVA showed a significant effect on the emotion of being moved at T1, *F*(2, 226)=60.89, *p*<.001, *η_p_* ^2^=.35. The art installation elicited higher levels of being moved, compared to the death control (*p*<.001) and the neutral control groups (*p*<.001). H1 is accepted. The difference between the death control and the neutral control groups was also significant (*p*<.001). See **Table 1**.

In line with H3a, the art installation elicited higher levels of connectedness at T1, compared to the death control (*p*<.001) and the neutral control groups (*p*<.001), *F*(2, 226)=43.62, *p*<.001, *η_p_* ^2^=.279. The difference between death control and the neutral control group was also significant (*p*<.001). See **Table 1**.

Both effects remained significant at the *p*<.001 level when including mental simulation as a covariate in the analyses. Mental simulation was a significant predictor of being moved (*F*(1,225)=60.59, *p*<.001, *η_p_* ^2^=.21), and of connectedness (*F*(1,225)=59.38, *p*<.001, *η_p_* ^2^=.21).

### T2 measurements

Of the 229 participants, 105 completed the second questionnaire that was distributed two weeks later, among which 52 from the art installation group, 26 from the death control group and 27 from the neutral control group. The response rate was thus lower in the two control groups, however there were no between-group differences regarding demographic variables (see **Table 1**).

The ANOVA on Lingering Reflection (*N*=105) revealed a significant effect of condition, *F*(1, 102)=14.43, *p*<.001, *η_p_* ^2^=.221. Participants from the art installation group had reflected significantly more about the ‘experience your own death’- project than participants from the death control g (*p<.*001) and neutral control group (*p*<.001), therefore H2 is supported. The control groups did not differ (*p*=.657). See **Table 2**. Including mental simulation as a covariate in the analyses showed that mental simulation and retrospective reflection were uncorrelated (*F*(1,101)=3.13, *p*=.080).

**Table 2:** Means (SDs) on Being Moved (T1), Connectedness (T1), and Reflection (T2) as a function of experimental condition (art installation, death control, neutral control).

	Being Moved (N=229)	Connectedness (N=229)	Reflection (N=105)
Art installation	4.48 (1.13)^a^	3.03 (0.81)^a^	2.83 (1.06)^a^
Death control	3.07 (1.27)^b^	2.24 (1.02)^b^	1.73 (0.67)^b^
Neutral control	2.29 (1.35)^c^	1.71 (0.82)^c^	1.85 (1.10)^b^

NB: Means with different superscripts (columns) differ significantly.

Because Life Appreciation was measured at both time points, we conducted a 3x2 Repeated Measures ANOVA (*N*=105) on Appreciation of Life (T1, T2) with condition as a between-subjects factor. G-Power sample size analysis for the repeated measure analysis with a power of.80 and an effect size of *f* =.25 revealed a required simple size of 81.

The analysis revealed a significant main effect for Time, *F*(1, 102)=4.375, *p*=.039, *η_p_* ^2^=.041, and a significant main effect for Condition, *F*(2, 102)=4.51, *p*=.013, *η_p_* ^2^=.081. Participants in the art installation (*M*=2.68, *SE*=.13; *p*=.004) and death control group (*M*=2.56, *SE*=.18; *p*=.040) appreciated their life more, compared with the neutral control group (*M*=2.03, *SE*=.18). Appreciation of life decreased over the course of two weeks for all groups (*M*= 2.59, *SD*=1.09 vs *M*=2.37, *SD*=1.10). The interaction was not significant (*F*<2, *p*>.18). Therefore, H3b is not supported. Including mental simulation as a covariate in the analyses showed a nonsignificant relationship between mental simulation and appreciation of life (*F*(1,101)=3.18, *p*=.077).

### Exploratory analyses: indirect effects

To explore the possibility that the experimental conditions affected Appreciation of Life at T2 indirectly, we entered Being Moved at T1, Connectedness at T1, Appreciation of Life at T1and Lingering Reflection at T2 as mediator of the effects of experimental condition on Appreciation at T2 in PROCESS Model 4 (24, 25), using Helmert contrasts (control groups vs. art installation; death control vs. neutral control).

The model confirmed an indirect path for Lingering reflection, in which the art installation increased Lingering Reflection at T2, which in turn increased Life Appreciation at T2, compared with the two control groups (CI -.528 to -.061). The indirect path was not significant for the comparison between the death and neutral control groups (CI -.098 to.172).

**Figure 2:**
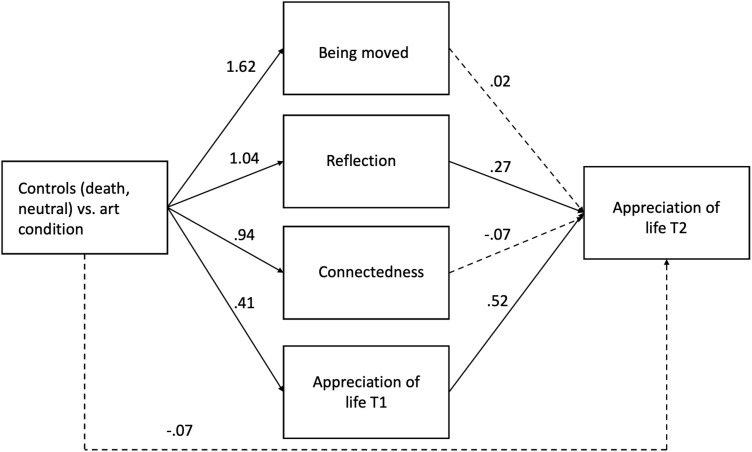
Indirect effects path from art installation (vs controls) on Appreciation of life at T2, via Appreciation of Life at T1 and Reflection at T2 (*N*=105). Dotted lines represent non-significant paths.

The analysis also confirmed an indirect path for life appreciation at T1 for the comparison between the art installation condition versus the two controls (death, neutral; CI -.464 to -.002). Thus, the art installation increased Life Appreciation at T1, which, in turn, significantly predicted Life Appreciation at T2. This indirect path was also significant for the comparison between the death and the neutral control group (CI -.819 to -.102), implying that the death control group also increased life appreciation at T1, which, in turn predicted life appreciation at T2. No indirect paths were observed for Being Moved and Connectedness at T1. See **Figure 2**.

## Discussion

In the present research we tested effects of an art installation that invited visitors to meditate on their own death. Findings showed that the art-induced death meditation emotionally moved visitors, made them feel part of a bigger whole, and increased lingering reflection and life appreciation until two weeks after exposure, compared with a death and neutral control condition. These findings point to the harnessing power of art in addressing difficult topics such as death. The fear of death is the most fundamental human fear that individuals typically deal with by suppress all thoughts of it (5). While death thought suppression may be a very common response to the fear of death, our findings show that other, more reflective responses to death are possible, and that art can play a role in eliciting such responses.

The findings suggest that there is sustained value in confronting one’s own death. Two weeks after their death meditation, visitors of the art installation showed lingering reflections about life and death, compared with the control groups. These lingering reflections, in turn, positively predicted individuals’ appreciation of their own life. Two findings rule against an alternative explanation of our findings, according to which increased life appreciation is just another cultural worldview defense strategy to suppress the fear of death. First, all participants were primed with death during recruitment in the same way, and there were no differences in implicit death thought activation across conditions. Second, death thought suppression typically occurs close after individuals have been reminded about death, but participants’ reflection about life and death extended well beyond immediate exposure to the death stimuli. Therefore, our findings appear indicative of a sustained growth response, much like those that have been documented for NDE (also see 26). Like individuals who experienced a NDE, visitors of the art installation showed increased connectedness with fellow humans, with nature and a higher power (20), and an increased appreciation of life (21).

The finding that the art installation emotionally moved visitors and increased lingering reflection confirms our premise that the art installation triggered a eudaimonic experience. Previous research has shown that eudaimonic entertainment can invite viewers to confront the fear of death at a safe aesthetic distance, by watching a meaningful story unfold through the eyes of the protagonist, while being safely seated on one’s own couch or in a movie theater (9). In a similar vein, visitors of the art installation experienced a beautiful and meaningful simulation of what death could be like, while being safely seated on their chair in a concert hall. Aspects of the art installation such as the concert hall, the carefully laid out field of bone dust, and the poetic words from the guided meditation may have provided visitors with the safe aesthetic distance to reflect on the difficult topic of death. Such a perspective is often lacking in the real world, where individuals avoid thinking and talking about death. The fact that participants in the death control condition, who reflected on death outside of the art installation, were not emotionally moved and did not show lingering reflection supports this argument.

While the death control group did not produce effects on being moved, a sense of connectedness to a bigger whole, and lingering reflection, it is interesting to note that it exerted similar effects as the art installation condition on life appreciation at T1, which, in turn, predicted life appreciation at T2 for both the art installation and the death control group. These findings suggest that typical efforts to manage the awareness of death may not only have adverse effects on individuals and society but may also instill adaptive responses. Other researchers have made similar observations, noting that mortality salience can, for example, motivate individuals to build supportive relationships and foster open-minded and growth-oriented behaviors (27, 28).

## Limitations and future directions

We cannot rule out self-selection effects in the sample, for example regarding specific death attitudes, or other factors that increase participants’ interest in the topic of death. Therefore, the present findings are not generalizable to the larger population. It is difficult to prevent self-selection biases for topics such as these, because asking participants to engage in an intensive death simulation without informing them about this activity beforehand would be unethical. Within the ethical boundaries that we needed to work with, we believe that our approach, in which we recruited all participants from the same sample of people interested in the topic, minimized systematic differences between the experimental groups. This was confirmed by the fact that there were no systematic differences between participants in the experimental and control groups on key demographic variables.

Another issue concerns the comparability of the different experimental conditions. In the present research we intentionally set out to compare the effects of the art installation to well-validated control conditions from TMT research. We build on previous TMT studies to operationalize the two control conditions, which were highly comparable in form and differed only on one dimension. In contrast, the art installation had a different form, which included verbal suggestions and visual experiences that the control conditions lacked. For this reason, we cannot ascertain which aspects of the art installation were vital to produce the observed effects, and which were negligible. For example, we cannot distill the specific effects of the aerosoled field of human bone dust, or the different stages of decay presented in the guided meditation. While technically it would be perhaps possible to ask artists to make comparable control versions that differed only on one dimension for the sake of science, this would not serve the purpose of the artist. Nevertheless, we do want to encourage future research to further look into these questions. Such research could also use a pre-posttest design to assess within participant changes over time before and after exposure to a guided death meditation.

There was a selection effect at follow-up; individuals from the installation group completed the T2 questionnaire more often than the two control groups. Although this can be regarded as a slight drawback in a methodological sense, it underscores the power of the art installation to evoke enduring inspired and engaged participants. The overlap between measures of connectedness used in eudaimonic entertainment research and the larger concept of posttraumatic growth, specifically the subscale of relating to others, is worth exploring further. Similarly, the overlap between the current findings and previous research on beneficial effects of meditation (29) and death reflection (30) is worth exploring further.

Our findings showed that the art installation elicited more lingering reflection two weeks after exposure than the two control groups, but they do not provide any insight into the depth of reflection or the exact content of participants’ lingering reflections. We therefore recommend future studies to include a depth of reflection measure and/or asking participant to write down reflective thoughts. Future studies should also attempt to retain a larger sample at follow-up, as our T2 sample was rather small to conduct mediation analysis (31).

## Concluding remarks

Our findings confirm ancient Buddhist and philosophical notions about the value of death reflection, showing that meditating on one’s own death can help individuals to connect to and appreciate life. The findings are relevant for healthcare contexts, inviting both patients and healthcare professionals to incorporate different, more nuanced images of death and dying in their daily practices. Several end-of-life practitioners already suggest movies to their patients and relatives to change perspectives, and the present findings underscore that not only movies, but also art can help individuals embrace the idea of death.

The findings are also relevant for modern society, showing that meaningful art can make room for death in a society that treats death as an existential problem that should be avoided rather than a natural part of the life cycle that should be discussed.

## Data Availability

The datasets presented in this study can be found in Radboud Data Repository, DOI 10.34973/hw0z-rd91 (https://doi.org/10.34973/hw0z-rd91).
